# Draft Genome Sequences of Two Glycoalkaloid-Degrading *Arthrobacter* Strains Isolated from Green Potato Peel

**DOI:** 10.1128/MRA.00226-19

**Published:** 2019-04-18

**Authors:** Rosanna C. Hennessy, Byeonghyeok Park, Duleepa Pathiraja, In-Geol Choi, Mikkel Schultz-Johansen, Peter Stougaard

**Affiliations:** a Department of Plant and Environmental Sciences, University of Copenhagen, Frederiksberg C, Denmark; b Department of Biotechnology, College of Life Sciences and Biotechnology, Korea University, Seoul, South Korea; Broad Institute of MIT and Harvard University

## Abstract

Here, we report the genome sequences of two *Arthrobacter* sp. strains isolated from potato and capable of degrading the toxic potato-derived glycoalkaloids (GAs) α-chaconine and α-solanine. Information from the genome sequences will provide insight into the genetic mechanism of GA degradation by these isolates.

## ANNOUNCEMENT

Glycoalkaloids (GAs) are chemical compounds consisting of an alkaloid attached to a carbohydrate moiety (1). The two main GAs produced in potato are α-chaconine and α-solanine. Potato juice is a by-product of starch processing and a potential protein source for human nutrition. To convert starch side streams from feed to food, antinutritional factors, including GAs, must be removed. While fungal pathogens are known to degrade potato-derived GAs, little information on bacterial enzymes is available (2). This announcement reports the draft genome sequences of two *Arthrobacter* strains (S39 and S41) which are capable of degrading α-chaconine and α-solanine.

Previously, the GA-degrading strains *Arthrobacter* sp. S39 and *Arthrobacter* sp. S41 were isolated from green potato peel (2). Genomic DNA was purified from liquid cultures grown at 20°C in lysogenic broth (LB) using the Gentra Puregene Yeast/Bact. kit (Qiagen, Germany) as previously described (3). Library preparation for whole-genome sequencing was performed using the Nextera DNA library prep kit (Illumina, Inc., San Diego, CA). Sequencing was carried out with a MiSeq platform using the MiSeq reagent kit v3 (600 cycles) (Illumina, Inc.). A total of 979,725 paired-end reads (589.8 Mbp) with an average read size of 301 bp and an average insert size of 525 bp were retrieved for *Arthrobacter* sp. S39, and a total of 1,376,771 paired-end reads (828.8 Mbp) with an average read size of 301 bp and an average insert size of 407 bp were obtained for *Arthrobacter* sp. S41. Adapter sequences and low-quality bases (<Q20) were trimmed from raw reads using TrimGalore! software v0.4.4 (https://www.bioinformatics.babraham.ac.uk/projects/trim_galore/). A total of 962,128 reads (405.8 Mbp, 79.3× coverage) for *Arthrobacter* sp. S39 and 1,363,873 reads (596.9 Mbp, 171.9× coverage) for *Arthrobacter* sp. S41 passed quality control and were used for assembly using the SPAdes genome assembler v3.7.1 with default settings (4). Genome annotation was conducted using the Prokaryotic Contig Annotation Pipeline Server (P-CAPS) (5) and the NCBI Prokaryotic Genome Annotation Pipeline (PGAP) (6). The annotation results of P-CAPS were not incorporated into the results of PGAP. P-CAPS was used to inspect the quality of the assembly prior to submission to NCBI.

The draft genome sequence of *Arthrobacter* sp. S39 is 5,118,715 bp long, and its contigs are contained in 48 scaffolds (*L*_50_, 5; *N*_50_, 391,678 bp) with a mean G+C content of 65.4%. The draft genome sequence of *Arthrobacter* sp. S41 is 3,472,078 bp long, and its contigs are set up in 7 scaffolds (*L*_50_, 1; *N*_50_, 2,465,108 bp) with a mean G+C content of 55.7%. The annotation of the *Arthrobacter* sp. S39 genome revealed the presence of 4,587 predicted coding sequences (CDS) and 72 predicted RNA coding sequences (18 rRNAs, 51 tRNAs, 3 noncoding RNAs [ncRNAs]), compared to 3,119 CDS and 72 RNA genes (4 rRNAs, 65 tRNAs, 3 ncRNAs) for *Arthrobacter* sp. S41.

Carbohydrate-active enzymes (CAZymes) were identified using a local version of dbCAN v6.0 (E value cutoff, 1.0e-5) (7). A total of 283 predicted CAZy domains were identified, including 141 glycoside hydrolases (GHs) in *Arthrobacter* sp. S39 and 39 GHs in *Arthrobacter* sp. S41. The genomes of both strains encode GHs organized in gene clusters containing α-l-rhamnosidases, β-d-glucosidases, and β-d-galactosidases, which are candidate enzymes for GA degradation with potential application in the removal of toxic GAs present in potato side streams for conversion into protein suitable for human nutrition.

### Data availability.

This whole-genome shotgun project has been deposited at DDBJ/ENA/GenBank under the accession numbers SIHX00000000 (*Arthrobacter* sp. S39) and SIHY00000000 (*Arthrobacter* sp. S41). The versions described in this paper are SIHX01000000 and SIHY01000000, respectively. Raw sequence reads are available under SRA accession numbers SRX5464543 (*Arthrobacter* sp. S39) and SRX5464544 (*Arthrobacter* sp. S41).
